# Roles of Gibberellin Catabolism and Signaling in Growth and Physiological Response to Drought and Short-Day Photoperiods in *Populus* Trees

**DOI:** 10.1371/journal.pone.0086217

**Published:** 2014-01-20

**Authors:** Christine Zawaski, Victor B. Busov

**Affiliations:** School of Forest Research and Environmental Science, Michigan Technological University, Houghton, Michigan, United States of America; New Mexico State University, United States of America

## Abstract

Survival and productivity of perennial plants in temperate zones are dependent on robust responses to prolonged and seasonal cycles of unfavorable conditions. Here we report whole-genome microarray, expression, physiological, and transgenic evidence in hybrid poplar (*Populus tremula × Populus alba*) showing that gibberellin (GA) catabolism and repressive signaling mediates shoot growth inhibition and physiological adaptation in response to drought and short-day (SD) induced bud dormancy. Both water deprivation and SDs elicited activation of a suite of poplar GA2ox and DELLA encoding genes. Poplar transgenics with up-regulated GA 2-oxidase (GA2ox) and DELLA domain proteins showed hypersensitive growth inhibition in response to both drought and SDs. In addition, the transgenic plants displayed greater drought resistance as evidenced by increased pigment concentrations (chlorophyll and carotenoid) and reductions in electrolyte leakage (EL). Comparative transcriptome analysis using whole-genome microarray showed that the GA-deficiency and GA-insensitivity, SD-induced dormancy, and drought response in poplar share a common regulon of 684 differentially-expressed genes, which suggest GA metabolism and signaling plays a role in plant physiological adaptations in response to alterations in environmental factors. Our results demonstrate that GA catabolism and repressive signaling represents a major route for control of growth and physiological adaptation in response to immediate or imminent adverse conditions.

## Introduction

Phenotypic plasticity in response to adverse conditions determines plant productivity and survival. Abiotic stress results in the largest loss in crop yields worldwide [1] and is a major threat to crop sustainability [2]. Thus, improving abiotic stress resistance is considered to be a main route for sustainable yield growth and will likely become progressively more important as arable land is becoming increasingly limited [3] due to (1) the deterioration of previously productive lands [4], (2) the predicted expansion of areas affected by droughts [5] and high salinity [6], and (3) the predicted increase in the occurrences of climatic extremes [7].

Plants reduce growth under adverse conditions as a mechanism to avoid potentially lethal stresses [8]. In addition, plants can utilize environmental cues to detect and anticipate imminent, adverse conditions and correspondingly adjust their growth [9]. For example, woody perennials (trees and shrubs) from temperate latitudes stop shoot growth in response to short-day (SD) photoperiods that signal the approaching winter and impending months of dehydration and freezing conditions. The cessation of shoot growth precedes a more permanent growth inhibition known as winter dormancy that can last months, requires development of a specialized organ (e.g., bud), and entails physiological resetting to allow resumption of growth [10].

Gibberellins (GAs) are involved in regulating several aspects of plant growth and development [11]–[13]. The GA metabolic and signaling pathways have been extensively studied. The GA 2-oxidases (GA2ox) and DELLA domain proteins, like GAI (GA-insensitive) and RGL1 (repressor of ga1-3 like), are important regulators of GA levels and signaling. GA2oxs are enzymes that catalyze the 2-oxidation inactivation of both bioactive GAs and some of their precursors [14]–[16]. Overexpression of GA2oxs in transgenic plants leads to bioactive GA-deficiency and various levels of dwarfism [15], [16]. GA2oxs are encoded by small gene families which regulate specific processes in plants, in part by specific expression patterns [15]–[18]. DELLA domain proteins are strong repressors of several GA responses and characterized by the conserved DELLA domain which mediates the susceptibility of the protein to proteolytic degradation [19]. Mutant forms of these proteins (*gai* and *rgl1*) with truncation of the DELLA domain are resistant to degradation and impart repressive blocks to several GA-mediated responses [20]–[22].

An accumulating body of evidence suggests that DELLA domain proteins and GA2oxs are involved in plant abiotic stress response. For example, activation of DELLA domain proteins appears to be crucial for restraining growth in adverse conditions [23]–[25]. DELLA proteins are believed to affect both cell expansion [11] and proliferation [26], [27]. For instance, DELLA proteins have been shown to inhibit cell proliferation through elevation of cell cycle inhibitors [27], and to promote cell differentiation by reducing inhibitors of the developmental transition from mitosis to endoreduplication that modulate anaphase-promoting complex/cyclosme activity [26]. DELLA proteins not only inhibit growth but also promote plant survival under stressful conditions by limiting the accumulation of reactive oxygen species (ROS), thus delaying cell death [25]. In Arabidopsis (*Arabidopsis thaliana*), salt stress leads to DELLA protein stabilization and as a consequence, growth inhibition and increased plant survival [23]. In addition, a DELLA protein in Arabidopsis has been shown to bind to the promoter and increase expression of the *XERICO* gene, which is involved in drought response [28]. DELLA proteins have also been implicated in mediating hormonal cross-talk between GA andabscisic acid (ABA) signaling pathways [23], [28]. ABA is a growth inhibiting hormone that regulates one of the two major stress signal transduction pathways in plants [29].

In addition to modulation of GA sensitivity, stressful conditions can directly influence levels of bioactive GAs. For example, cold treated Arabidopsis plants have been shown to have increased expression of three *GA 2-oxidase* (*GA2ox*) genes [24], whereas under salinity stress six *GA2ox* genes were shown to be up-regulated [30]. Furthermore, the cold-inducible CBF1/DREB1b protein in Arabidopsis imparts freezing tolerance, at least in part by activating the expression of *GA2ox* genes, which in turn leads to reductions in bioactive GAs and suppression of growth [24]. Similarly, in Arabidopsis the DWARF AND DELAYED FLOWERING 1 (DDF1) protein, involved in salt stress response, binds to the promoter and activates the *GA2ox7* gene [30]. Though GA2oxs’ role in control of seed dormancy has been well substantiated [31], [32], their involvement in regulation of winter bud dormancy is based solely on correlative evidence. For instance, in several tree species, SD-induced transition to dormancy is associated with reduction in bioactive GAs [33]–[35].

Changes in GA catabolism and signaling can have profound effects on tree growth, phenology, morphology, physiological, metabolism, and gene expression [36]–[39]. In *Populus*, GA-deficient and GA-insensitive transgenics are semidwarfs of varying degrees of severity [39]. Semidwarfism has also been associated with other characteristics that could be advantageous under adverse conditions such as increased biomass allocation to roots, reduced stem elongation, and increased water use efficiency [40]. The wide diversity of effects associated with GA modulation suggests that GA metabolism and signaling could be involved in mediating plant adaptive responses to adverse environmental conditions. Here, using a diverse array of evidence, we show that both DELLA and GA2ox encoding genes in hybrid poplar (*Populus tremula x Populus alba*) constitute a major regulatory circuit mediating growth restraint and physiological adaptation to drought stress and SD photoperiods.

## Results

### Poplar DELLA Domain and GA2ox Encoding Genes are Induced by Drought and SDs

**Figure 1:**
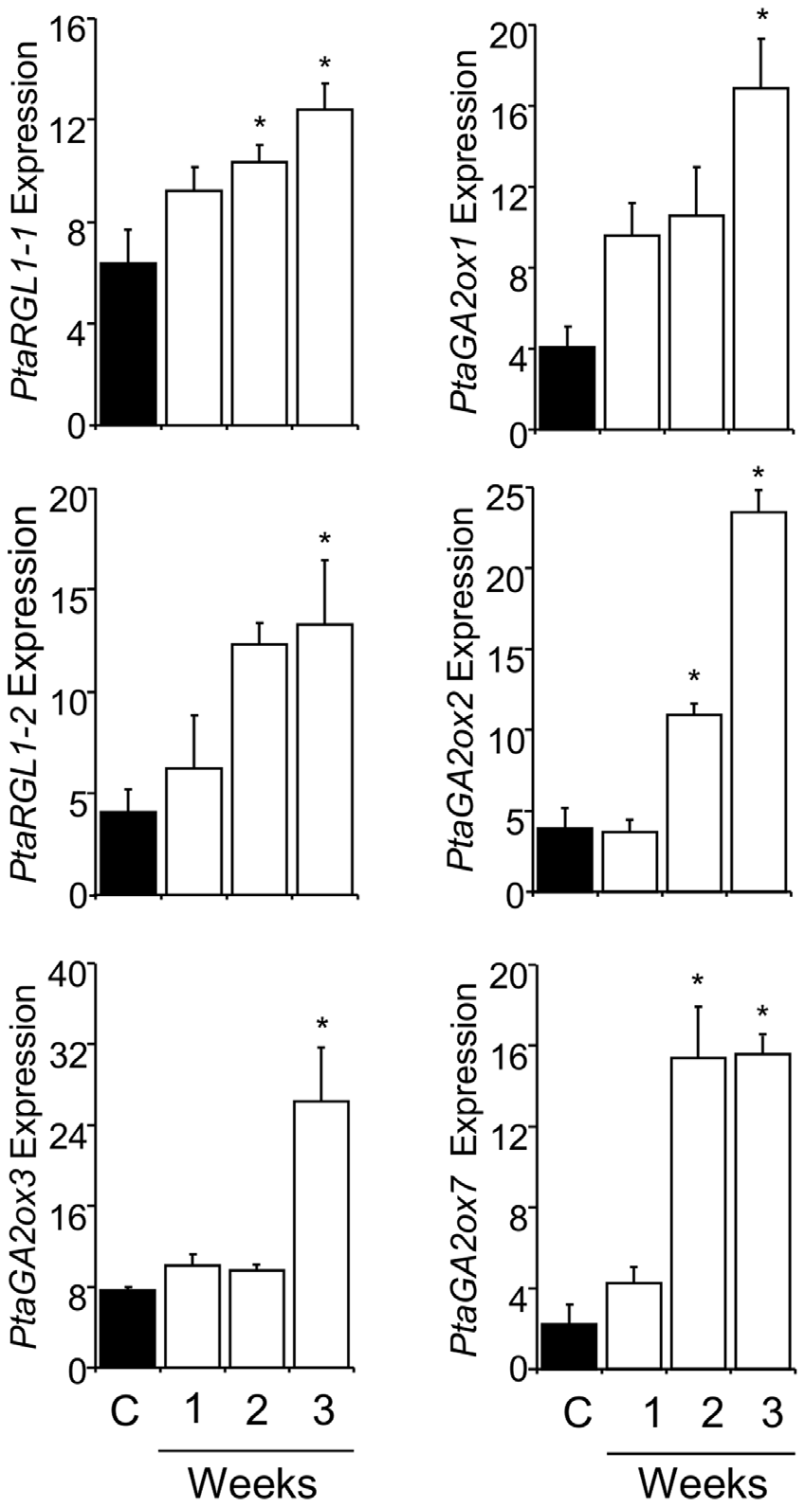
Poplar DELLA domain and GA2ox encoding genes were significantly up-regulated in response to drought stress. Shown are mean±SE of RT-PCR results for three biological reps each consisting of leaf tissue pooled from 2–3 plants for well-watered control (C) and water-withholding (1–3 weeks) treatments. Expression was normalized to *Ubq* and *Cyc.* Significant differences between watered and water-withholding treatments were determined by one-way ANOVA followed by Dunnett’s post-hoc test (*, P<0.05).

**Figure 2:**
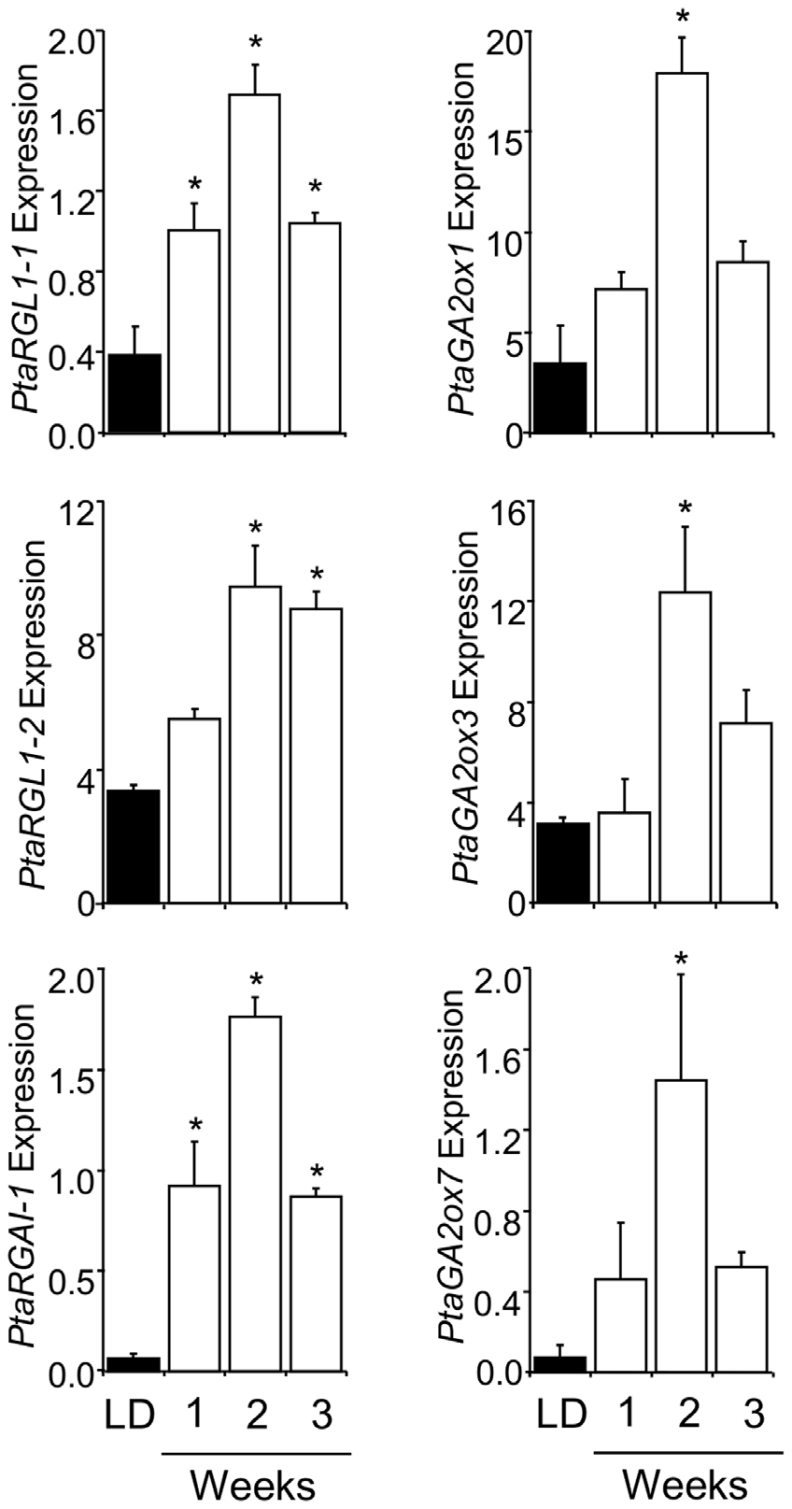
Poplar DELLA domain and GA2ox encoding genes were significantly up-regulated by SD photoperiod. Shown are mean±SE of three biological reps each consisting of leaf tissue pooled from three plants subjected to long-day (LD) and SD (1, 3, and 5 weeks) treatments. Expression was normalized to *Ubq* and *Cyc*. Significant differences between LD and SD treatments were determined by one-way ANOVA followed by Dunnett’s post-hoc test (*, P<0.05).

We studied expression of four poplar DELLA protein (*PtaGAI1*, *PtaGAI2*, *PtaRGL1-1*, and *PtaRGL1-2*) and seven PtaGA2ox (*PtaGA2ox1* to *7*) encoding genes (Table 1) in leaves in response to drought and SD photoperiods (Figures 1 and 2). Drought treatment, imposed through water deprivation under greenhouse conditions, increased expression in two of the four DELLA protein encoding genes and four of the seven *PtaGA2ox* genes (Figure 1). Expression increased weekly, reaching peak levels for most genes at the end of the studied period. The largest increase in expression occurred for *PtaGA2ox2* and *PtaGA2ox7* which showed seven-fold induction (Figure 1). To study the role of the same genes in growth cessation during SD-induced bud dormancy, we imposed a SD photoperiod (8 h light/16 h dark) under controlled growth chamber conditions (see Materials and methods) and followed changes in expression in the leaves on a weekly basis. Expression of three of the four DELLA protein encoding genes and three of the seven *PtaGA2ox* genes increased significantly (Figure 2). There was substantial overlap in the expression of genes up-regulated by both drought and SDs (*PtaRGL1-1*, *PtaRGL1-2*, *PtaGA2ox1*, *PtaGA2ox3*, and *PtaGA2ox7*). Expression of only two (*PtaGAI1* and *PtaGA2ox2*) of the seven genes was specifically influenced by one but not the other treatment (Figures 1 and 2).

**Table 1:** Names, models, and primer sequences for genes used in expression analysis.

		Primers (5′ to 3′)	
Name	Model	Forward	Reverse
*PtaGA2ox1*	Potri.001G378400	TGAGATTCTTGAAATGATGGCT	GCCTATTATCAGCCAATCTGGAG
*PtaGA2ox2*	Potri.002G191900	TGATGGTAAGGGCATGTGAAG	TCAAGATTTGAGGGTCGGAGT
*PtaGA2ox3*	Potri.004G065000	GCTGATCCCCTGAAACCAAGGA	TCAAATAGCCCAAGTCTGTAATCAGCTAGC
*PtaGA2ox4*	Potri.008G101600	ATGGTAGTGGCATCTCCAAC	TCATCGAATGGTTTGGCA
*PtaGA2ox5*	Potri.010G149700	ATCTGATGGCAGAGGGATTG	GTTAGGGCTTTGTGCCTCAC
*PtaGA2ox6*	Potri.011G095600	AATGATTTATTTTGGTGGACCAC	TAAACCTATTCTTGAAGTATTTGGCAAG
*PtaGA2ox7*	Potri.014G117300	TATTCAGTAGGCTCATCAGAGACG	GATTTTGCTGTGAAACCATGTG
*PtaRGL1_1*	Potri.004G089800	TCATGGGTTGAAGATGATCAAG	CACTCCTTGGACAACCTTCC
*PtaRGL1_2*	Potri.017G125200	GGTGGTGCTGGGAATTCT	TCAAATCCTTCCACAATGACC
*PtaGAI1*	Potri.008G131700	CACCGAGTCTGTGGCTGTT	CAAATTATGCCCTCAAAACTCACT
*PtaGAI2*	Potri.010G110700	TTATACCCTCAAAATTCAACCGA	TACTGAGTTCGAGTCTGTGGCT
*PtaCYC*	Potri.004G168800	GGCTAATTTTGCCGATGAGA	ACGTCCATCCCTTCAACAAC
*PtaUBQ*	Potri.002G224700	AGAGTGTGAGAGAGAGAAGAG	CGACGACCATCAAACAAGAAG

Gene models are according to poplar genome v.3.

### GA-deficiency and GA-insensitivity Accelerates Growth Inhibition in Response to Drought and SDs

**Figure 3:**
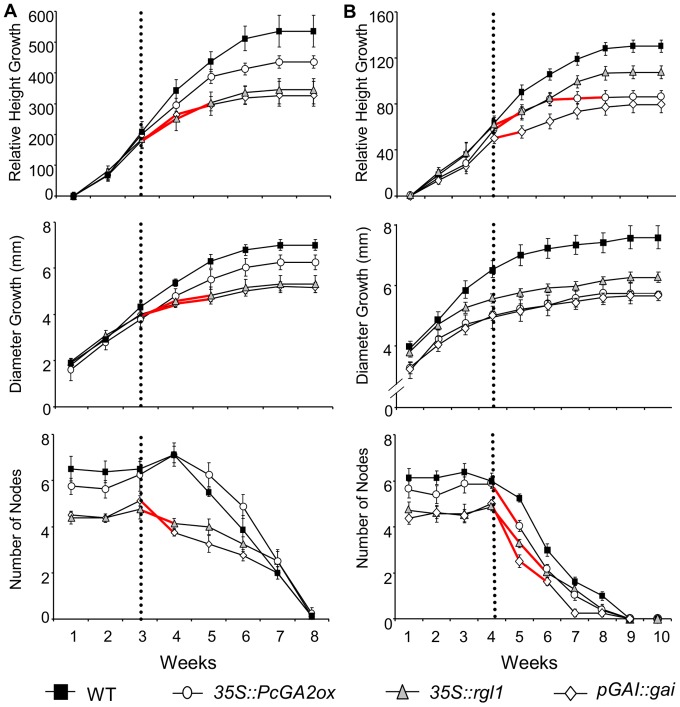
GA-insensitive and GA-deficient poplar ceased growth faster in response to drought and SD. Shown are weekly responses of transgenic and WT *Populus* subjected to drought stress (**A**) and SD photoperiod (**B**). Relative height growth is the percent increase in height from the initial height measured at the beginning of the experiment. The dotted line denotes the initiation of (**A**) water-withholding and (**B**) SD photoperiod. Red lines show significant differences between weekly relative growth rate of transgenics and WT (see Material and Methods for more details), as determined by one-way ANOVA followed by Dunnett’s post-hoc test (P<0.05).

The induction of *DELLA* and *GA2ox* genes in response to drought and/or SDs suggests that these genes may mediate growth inhibition during both responses. We took advantage of previously, well-characterized, GA-deficient (*35S::PcGA2ox*) and GA-insensitive (*35S::rgl1* and *pGAI::gai*) transgenic poplar with representative, stable, and intermediate/semidwarf phenotypes (see Materials and methods) to study their response to drought stress and SD photoperiods. To ensure that any inherent differences in size between genotypes did not obscure analysis of treatment effects, relative growth rates were used to determine significant differences between WT and transgenic plants. Experiments (drought and SD photoperiod) with transgenic plants were performed in a similar fashion to that of expression analysis and as described in the Materials and methods. Prior to implementing drought and SD photoperiod experiments, weekly relative growth rates were not significantly different between transgenic and WT plants under well-watered conditions and long-day photoperiods (Figure 3). For the drought experiment, to further facilitate valid comparisons between different genotypes, methods recommended by Verslues et al. [41] were employed; whereby, transgenic and WT plants were grown in the same pots so that roots of all genotypes would grow into the same soil and be exposed to similar conditions (see Materials and methods) (Figure S1). Because of the leaves’ importance in controlling water loss and previous results of transgenic genotypes having specific effects on leaf size [39], we measured leaf area and expansion (Figure S1). In support of our previous findings [39], the leaf area of GA-deficient transgenics was significantly different than WT, whereas GA-insensitive transgenics was not. However, expansion rates of newly formed leaves were similar between all transgenics and WT throughout the experiment (Figure S1). After only one week of withholding water, the *gai* and *rgl1* expressing transgenic plants had significantly reduced weekly relative growth rates in height, diameter, and number of nodes compared to WT (Figure 3A). Three weeks post-water deprivation, growth was virtually absent in *gai/rgl1* expressing plants whereas WT, and to a lesser extent *GA2ox* expressing plants, did not completely cease growth until weeks five and six. Interestingly, water deprivation also affected secondary woody growth (stem diameter at the base) in the *gai/rgl1* transgenics, as indicated by their significantly decreased growth rates relative to WT in weeks three through five (Figure 3A).

We also studied transgenics’ growth response to SD photoperiods that induce winter dormancy. The first response to SDs, which precedes and is a prerequisite for dormancy, is cessation of shoot growth. Poplars are highly photoperiod sensitive, and the genotype under investigation, *P. tremula x alba* (717 1B4), cease growth after three to five weeks under SD photoperiod [42]. All transgenics had significantly greater, early reductions in weekly relative growth rate compared to WT plants (as early as one week under SD) (Figure 3B). WT plants had a more gradual reduction in weekly growth and, as in the drought experiment, did not completely cease growth until the fifth week under SD conditions. In contrast to drought, we did not observe any differences between transgenics and WT with respect to reduction of diameter growth under SD conditions. Despite differences in growth cessation, the timing of bud set was not significantly (P>0.05) affected and occurred around week five in both transgenics and WT (data not shown).

### Physiological Changes in Response to Drought Stress

Drought stress has a profound effect on a number of physiological parameters in plants, and measurement of these parameters is useful in determining their stress response and resistance [43], [44]. To test if the *GA2ox* and *DELLA* expressing transgenic plants differ with respect to their physiological responses to drought, we measured several important parameters before and during drought stress response. We quantified pigment concentrations, photosynthetic rate, transpiration, and stomatal conductance, which are frequently used to measure the degree of stress imposed on plants (Table 2). In general, GA-insensitive and GA-deficiency had similar effects on measured responses (Table 2). Under well-watered conditions, all transgenics had significantly higher photosynthetic rates compared to WT (Table 2). Increases in photosynthetic rate corresponded to significantly higher total chlorophyll and carotenoids for all transgenic types (Table 2). Typically, drought stress reduces photosynthesis and causes degradation of chlorophyll. In contrast to WT, photosynthetic rates, chlorophyll and carotenoids remained significantly higher in all transgenics under drought conditions (Table 2). Under well-watered conditions transpiration and stomatal conductance were similar in transgenic and WT plants (Table 2). However, under drought stress, transgenic plants displayed significantly higher transpiration and stomatal conductance (Table 2). Transgenics had significantly higher water use efficiency under well-watered conditions as compared to WT. Under drought stress all transgenics showed a slight decrease in water use efficiency as compared to WT but only those expressing *gai* were significantly different (Table 2).

**Table 2:** Physiological changes in GA-deficient and GA-insensitive poplars under well-watered and drought conditions.

Condition	Genotype	Total Chlorophyll(mg g^−1^)^a^	Chlorophyll a/b ratio^a^	Carotenoid(mg g^−1^)^a^	Photosynthesis Rate (µmol m^−2^s^−1^)^b^	Transpiration Rate(mmol m^2^s^−1^)^b^	Stomatal Conductance(mol m^−2^s^−1^)^b^	Water Use Efficiency(µmol mmol^−1^)
Watered	WT	1.28±0.05c	1.96±0.12a	0.24±0.01c	12.32±0.22b	6.00±0.29a	0.32±0.07ab	2.08±0.07b
	*35S::GA2ox*	2.37±0.09a	1.68±0.16ab	0.36±0.01a	15.65±0.56a	6.18±0.29a	0.39±0.07ab	2.51±0.06a
	*35S::rgl1*	1.63±0.03b	1.70±0.11ab	0.26±0.01b	13.98±0.33a	5.68±0.16a	0.35±0.06a	2.44±0.06a
	*pGAI::gai*	1.67±0.05b	1.38±0.10b	0.26±0.01b	14.07±0.17a	5.92±0.36a	0.35±0.07ab	2.54±0.14a
Drought	WT	1.55±0.03b	1.94±0.11a	0.28±0.01b	6.56±0.64c	1.73±0.18b	0.07±0.01b	3.93±0.17a
	*35S::GA2ox*	1.99±0.07a	1.96±0.12a	0.34±0.01a	10.3±0.45ab	2.97±0.3a	0.17±0.02a	3.72±0.20ab
	*35S::rgl1*	1.98±0.05a	1.94±0.17a	0.32±0.01a	8.44±0.65b	3.18±0.48a	0.20±0.04a	3.12±0.28ab
	*pGAI::gai*	2.12±0.06a	1.36±0.06b	0.33±0.01a	9.05±0.39ab	3.01±0.39a	0.18±0.04a	3.36±0.22b

Means±SE are based on at least three biological replications. Well-watered and drought conditions were analyzed separately. Statistically significant differences were determined by one-way ANOVA followed by Tukey’s post-hoc test (P<0.05). Letters following mean±SE indicate statistical significance, where treatment (i.e., Genotype) means with the same letter are not significantly different.

^a^ Drought measurements 5 weeks after withholding water.

^b^ Drought measurements 3 weeks after withholding water.

**Figure 4:**
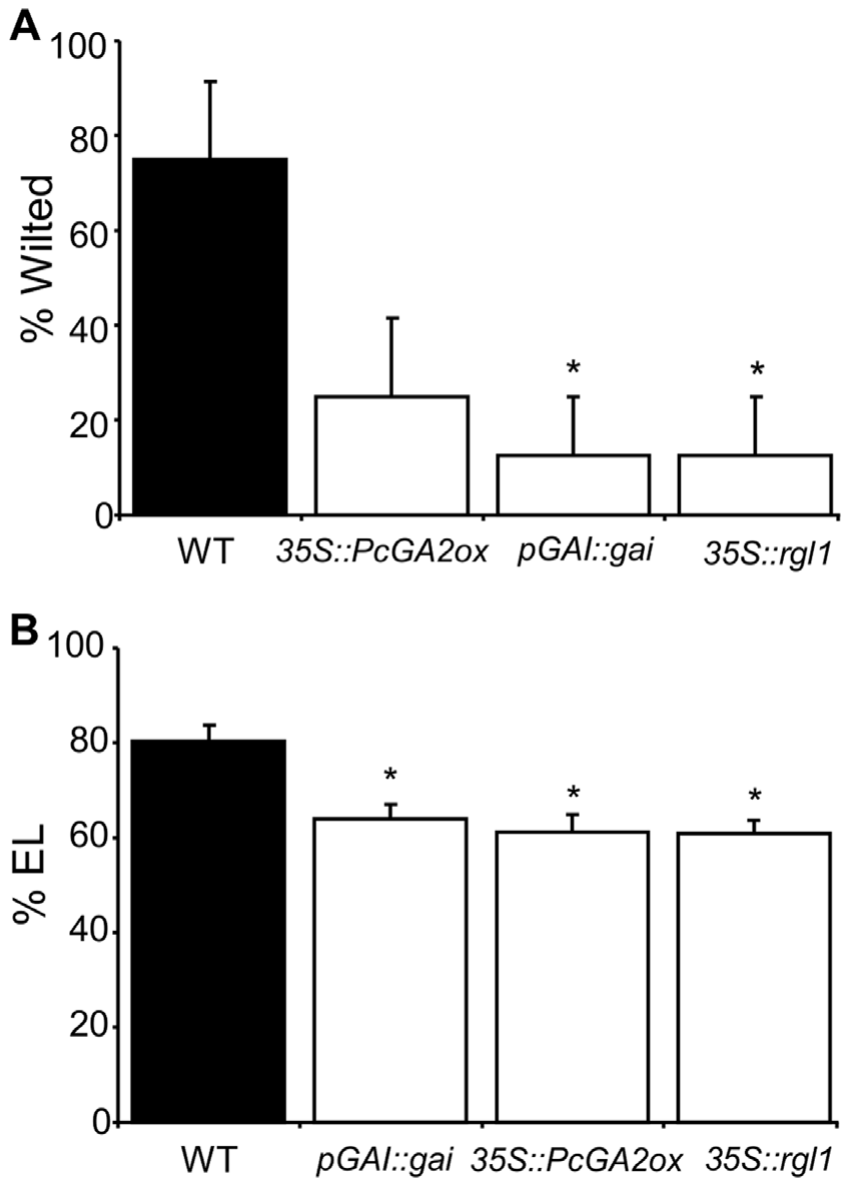
GA-deficient and GA-insensitive transgenics were more drought-tolerant than WT. Measurements were taken five weeks after water withholding on eight ramets/line and eight WT. (**A**) Significant differences between transgenic and WT were determined by Fisher’s exact test. (**B**) Significant differences between transgenic and WT treatments were determined by one-way ANOVA followed by Dunnett’s post-hoc test (*, P<0.05). EL = electrolyte leakage.

To assess the overall stress resistance of the different genotypes, we measured wilting and electrolyte leakage (EL). The *DELLA* expressing transgenics showed significantly lower levels of wilting compared to WT (Figure 4A). The GA2ox transgenics also showed a lower level of wilting but the differences were not statistically significant (P>0.05) (Figure 4A). EL can be used to quantify the extent of cellular damage caused by stress [41]. We found significantly lower EL in all transgenics (Figure 4B).

### Delayed Senescence in GA-deficient and GA-insensitive Poplars

**Figure 5:**
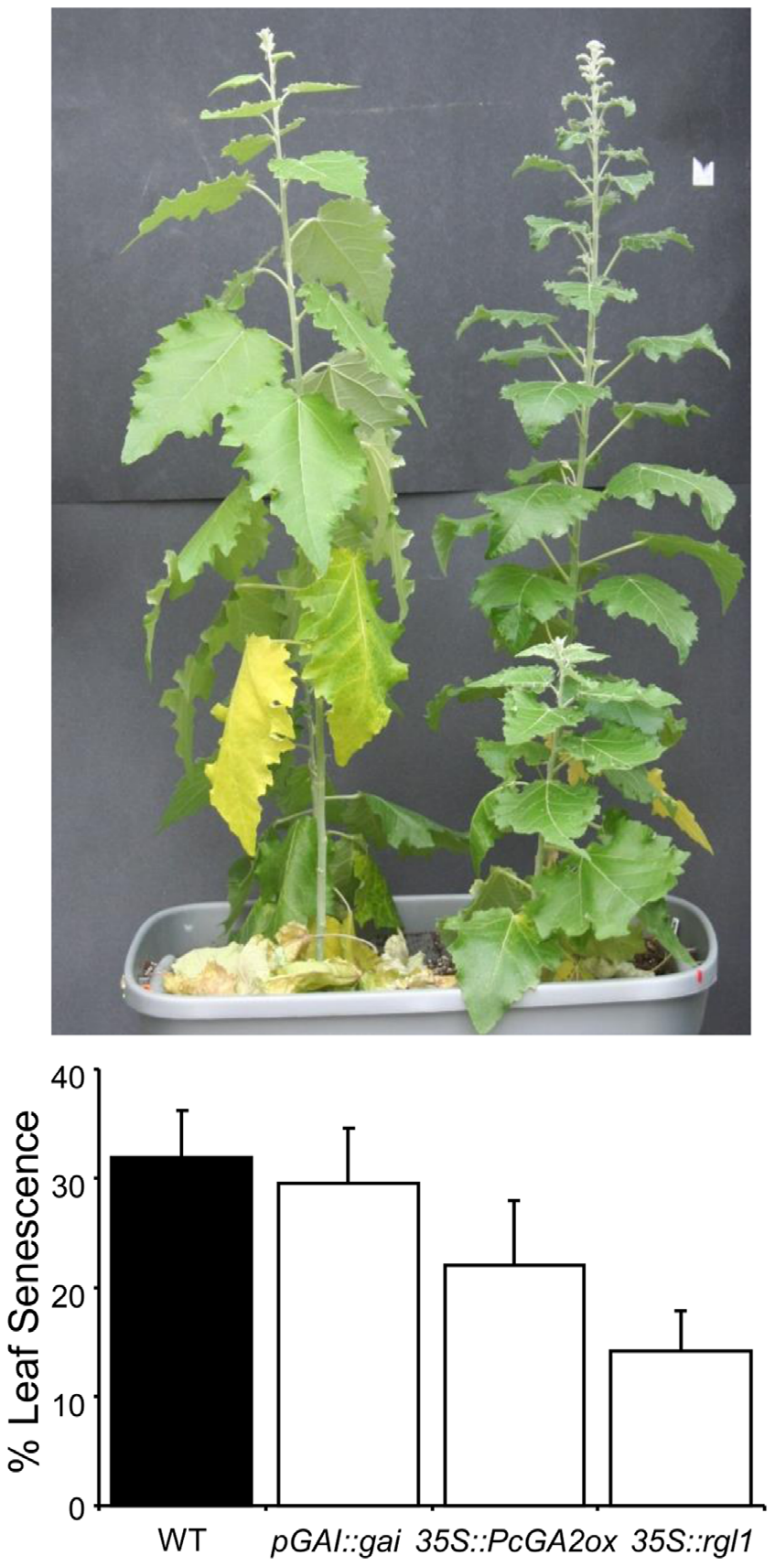
GA-modified transgenics displayed delayed senescence in response to drought stress. Representative picture shows WT (on left) and transgenic (*35S::PcGA2ox*) plants taken four weeks after water withholding. Measurements were taken five weeks after water withholding on eight ramets/line and eight WT.

In support of our quantitative pigment measurements, signs of senescence after water deprivation were more visible in WT leaves compared to transgenics (Figure 5). Percent of senescing leaves was not significantly different, but an apparent trend of more advanced senescence in WT as compared to transgenics was evident (Figure 5).

**Figure 6:**
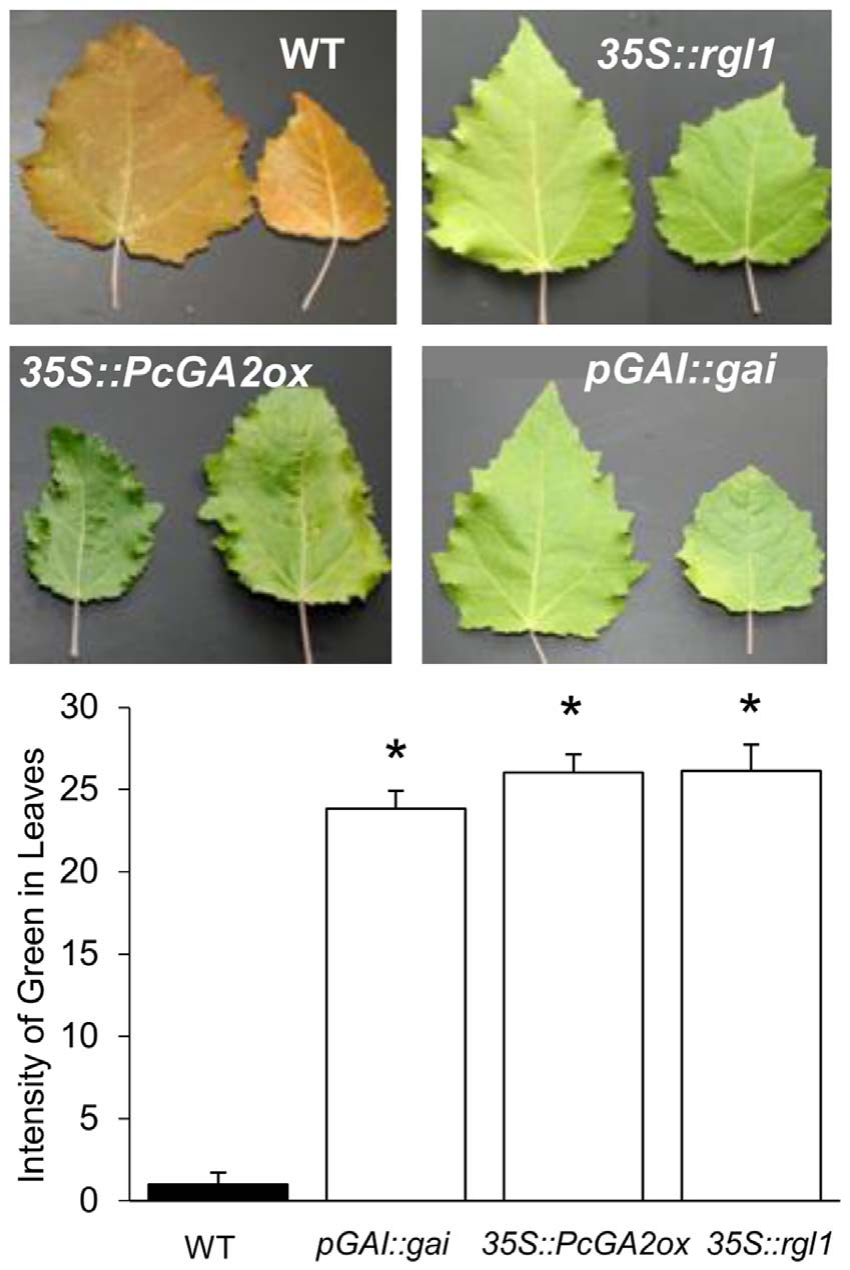
Delayed leaf senescence in GA-deficient and GA-insensitive poplar transgenics during SD-induced transition to bud dormancy. Two representative leaves are shown for comparison. Measurements represent computer based quantification of the relative intensity of green color in senescing leaves (see Material and Methods). Plants were grown for nine weeks under SD photoperiod (8 h). The temperature was maintained at 21°C for the first six weeks and reduced to 4°C for the remaining three weeks. Significant differences between transgenic and WT treatments were determined by one-way ANOVA followed by Dunnett’s post-hoc test (*, P<0.05).

Leaf senescence preceding winter dormancy is typically initiated by SD photoperiods but is enhanced by low temperatures (<10°C). To investigate senescence in GA-deficient and GA-insensitive plants, we subjected transgenic and WT plants to six weeks of SDs at 21°C followed by three weeks at 4°C and measured the extent of leaf senescence. In a manner similar to their response to drought, all transgenic plants showed delayed senescence when compared to WT (Figure 6).

### Increased Axillary Shoot Outgrowth in GA-deficient and GA-insensitive Poplars

**Figure 7:**
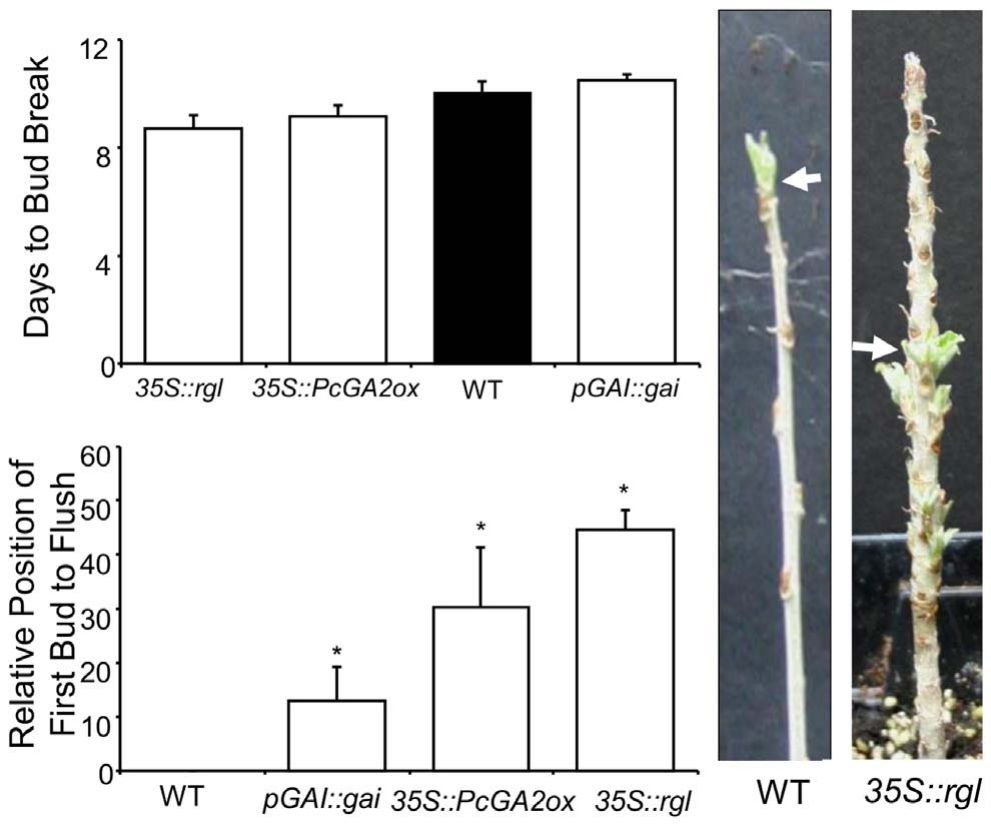
Bud flush in transgenic and WT *Populus.* Arrows point to the first bud to flush. Bars indicate mean±SE of at least three biological replications. Relative position of the first bud to flush was expressed as percent of buds from the top of the plant, 0% being the apex of the plant and 100% being the very bottom bud (closest to the soil). Significant differences between transgenic and WT treatments were determined by one-way ANOVA followed by Dunnett’s post-hoc test (*, P<0.05).

As with bud set, there were no significant differences in the timing of the first bud to flush after winter dormancy between transgenics and WT (Figure 7). However, in WT plants apical buds were always the first to initiate growth, whereas in transgenics axillary lateral buds typically flushed first (Figure 7).

**Figure 8:**
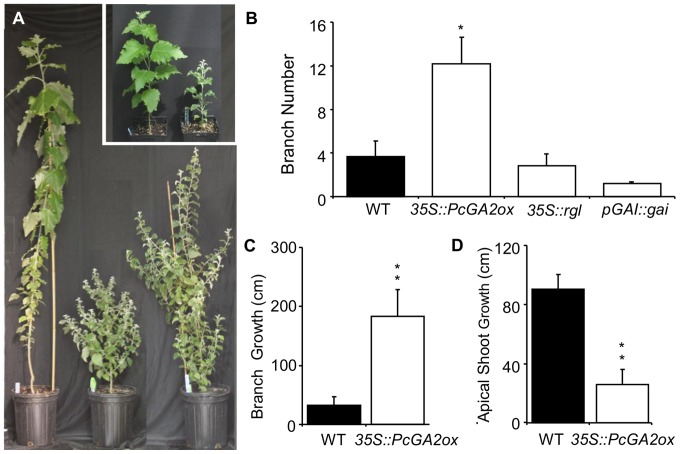
Decreased apical dominance in the GA-deficient (*35S::PcG2ox*) plants. (**A**) WT are on the left and *35S::PcGA2ox* plants are on the right. Inset shows plants prior to entering dormancy for comparison purposes. In (**B)** to (**D)**, bars represent mean±SE of eight ramets/line and eight WT. Statistically significant differences between WT and transgenic plants were determined by t-test (*P<0.05, and **P<0.01).

In *GA2ox* overexpressing transgenics, this was followed by a significant increase in lateral branch outgrowth as compared to WT plants (Figure 8A and B). In contrast to GA2ox transgenics, the flushed axillary buds in the DELLA expressing plants never elongated and remained in a leaf rosette stage. Because of the significant increase in lateral branches in GA2ox transgenics, we measured total height and branch growth three months after bud flush. Although GA2ox transgenics were significantly shorter, their branch length was significantly greater compared to WT (Figure 8C and D).

### Differential Expression of a Large Number of Genes in GA-modified Transgenics

Because the GA-modified transgenics showed accelerated response to drought and SDs, we hypothesized that the mechanisms associated with these responses are constitutively elevated even under control conditions (well-watered and long-day photoperiods). We therefore used whole-genome poplar microarray to compare transcriptomes of transgenic and WT leaves from plants grown under a control environment. We found 2,890 differentially expressed genes (ANOVA, P<0.01) (Table S1). Gene ontology (GO) analysis was used to gain insight into the global patterns of gene expression. Consistent with our results showing increased resistance to drought stress, we found ‘response to stress’ (GO:0006950) in the top 10 most significantly-enriched biological categories in the transgenic plants (Table S2). Among the genes associated with stress response were orthologs of CBF1 and CBF3 that encode AP2/ERF type transcription factors, and previously, CBF1-mediated cold stress response was shown to involve reductions in bioactive GA through increased *GA2ox* expression, which promoted DELLA protein accumulation [24].

Likely because GA is highly integrated into the regulatory network of other hormones [45], we found that at least one aspect of each of the five major hormones (e.g., auxin, cytokinin, brassinosteroid, ABA, ethylene) was significantly affected in the GA-modified transgenics (Table S2). GO categories associated with ethylene were prevalent in a number of categories and enrichment significance (Table S2). The trends in expression of these ethylene-associated genes are indicative of increased production and signaling through the ethylene signal transduction pathway. For example, genes encoding biosynthetic enzymes, such as acetyl-CoA carboxylase and acetyl-CoA synthetase, were up-regulated while the catabolizing ETHYLENE OVERPRODUCER 1 was down-regulated. Furthermore, downstream AP2/ERF type transcription factor genes were also up-regulated.

GA metabolism effects both cell expansion and proliferation [11]. The growth restraining effects of DELLA proteins has been shown to be, at least in part, due to regulation of cell proliferation [26], [27]. Previously, Achard et al. [27] showed that DELLAs restrain cell production by causing up-regulation of cell cycle inhibitors. We found the Kip-related protein (KRP3), a negative regulator of cell division, to be up-regulated in both GA-modified transgenics (Table S1), which is suggestive of a common mechanism for growth restraint through inhibition of cell proliferation.

### GA-deficiency and GA-insensitivity Share Common Transcriptome Responses with Dormancy and Drought

**Figure 9:**
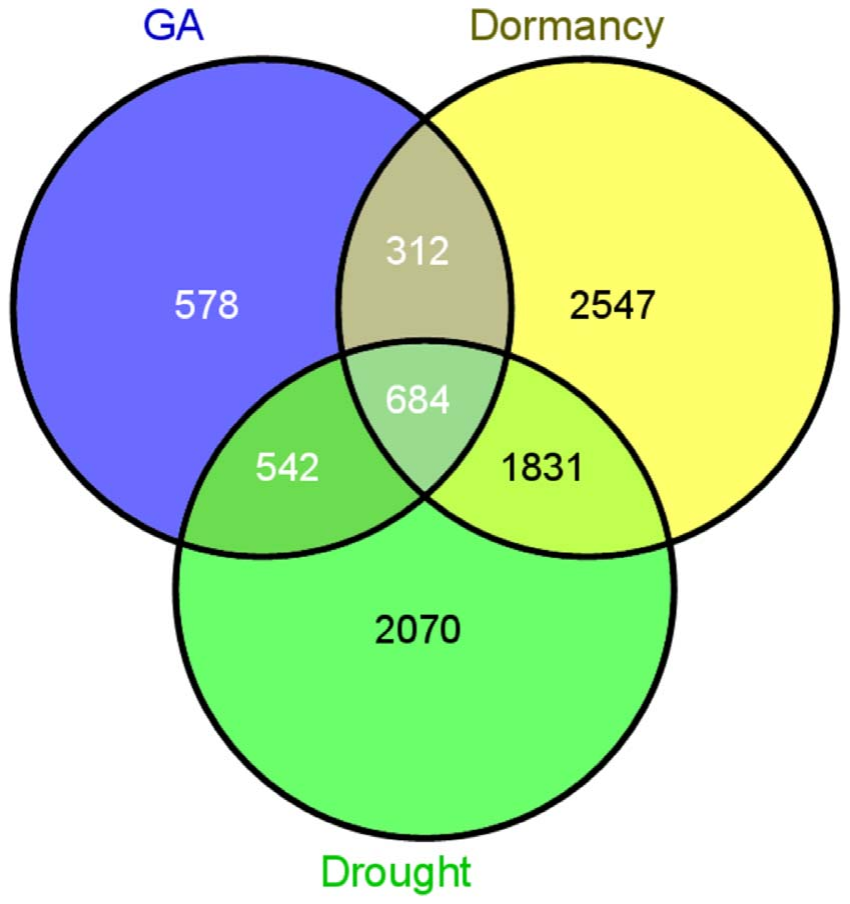
Significant overlap between GA-deficient and GA-insensitive (GA), dormancy, and/or drought transcriptomes. See text for details.

Because our studies suggest that GA-insensitive and GA-deficient plants have faster and more robust responses to dormancy-inducing conditions and drought, we speculated that alterations in GA metabolism and signaling in transgenics may result in transcriptome level changes that are shared with plants that are responding to stress (i.e., SD photoperiod and drought). Therefore, we compared the transcriptome of GA-deficient and GA-insensitive poplars to previously published transcriptomes of WT poplars during dormancy induction and drought response [46], [47]. Unfortunately, the data for dormancy induction was based on apices; microarray data for expression changes in poplar leaves was not available. Nevertheless, transcriptome comparisons could be useful in identifying differentially expressed genes that are not tissue-confounded. Indeed, nearly a quarter of all differentially expressed genes in the GA-modified transgenics were common with the genes found to be regulated during both dormancy induction and drought (Figure 9). In addition, the transgenics’ transcriptomes shared additional overlaps with either dormancy induction or drought. Among the most significantly enriched GO terms in the common transcriptome were processes associated with chloroplast biogenesis and function (Table S3).

## Discussion

Survival and productivity of long-lived perennial plants in temperate zones are dependent on robust responses to prolonged and seasonal cycles of unfavorable conditions. Here we report whole-genome microarray, expression, physiological, and transgenic evidence suggesting that GA catabolism and repressive signaling mediate shoot growth inhibition in response to both drought and SD-induced bud dormancy. Both water deprivation and SDs elicited up-regulation of a suite of poplar GA2ox and DELLA protein encoding genes (Figures 1 and 2). We also found that the two environmental factors elicited similar responses in the same set of genes with the only exception being *PtaGAI1* and *PtaGA2ox2*, which were up-regulated by one but not the other factor. Particularly instructive are the trends in expression of the *GA2ox* genes, for which we have performed a significant amount of previous work [17], [18]. For example, we found that *GA2ox3* was up-regulated by both drought and SD photoperiods (Figures 1 and 2). *PtaGA2ox3* is predominantly expressed in the apex, which contains the shoot apical meristems (SAM); furthermore, it was the only gene for which we failed to regenerate RNAi-suppressed transgenic plants, strongly suggesting that this gene is involved in organization of SAM [18]. Because growth, and particularly primary growth which originates in SAM, is most affected by drought and SDs, we believe that the up-regulation of *PtaGA2ox3* is likely an important mechanism for controlling growth through modulation of SAM activity. Another gene that was up-regulated by both drought and SDs was *PtaGA2ox7*. We have previously found that *PtaGA2ox7* and its paralog *PtaGA2ox2* are predominantly expressed in roots under optimal conditions and positively regulate lateral root proliferation [18], [37]. The fact that they are up-regulated by drought in leaves suggests they are involved in a mechanism for coordination of shoot-root ratio in relation to resource availability. Decreased aerial growth with increased lateral root growth would produce a plant that demands less of limited resources while still actively exploring the soil environment.

The commonalities in the transcription responses to drought and SDs are interesting because the signaling mechanisms induced by these two types of environmental cues are quite different. One involves a response to osmotic stress [48], while the other encompasses the light signaling pathway [49]. This suggests that GA2ox and DELLA protein genes are downstream of different signal transduction pathways that converge on the same types of genes. One possible convergence point could be through cross-talk with ABA biosynthesis or response. For example, DELLA proteins have been shown to regulate *XERICO*, which encodes a ubiquitin-ligase that positively regulates ABA biosynthetic encoding genes [28]. Indeed, our microarray work showed that a poplar ortholog of *XERICO* was highly up-regulated in the GA-modified transgenic plants. Alternatively the DELLA proteins themselves could be a convergence hub, as an increasing body of evidence suggests that they are focal points of multiple signaling pathways in plants [50]. Finally, ethylene can also play a role in the cross-talk, as our microarray results indicate a strong up-regulation of genes involved in ethylene biosynthesis and response.

Using previously characterized GA-deficient and GA-insensitive transgenics, with increased expression of GA2ox and DELLA protein encoding genes and representative semidwarf phenotypes [36], [39], [40], we show that GA-modified transgenics have significant changes in growth and physiological responses under both drought and SDs. Overall, overexpression of GA2ox and DELLA proteins in poplar caused hypersensitive growth inhibition under both drought and SDs. Relative growth rates were not significantly different between transgenic and WT plants under well-watered conditions and long-day photoperiods. In comparison, transgenics generally responded to water withholding and SDs by reducing relative growth on average one to three weeks earlier than WT (Figure 3). Nevertheless, traits were affected differently under drought and SD conditions. Decrease in GA concentration and sensitivity in transgenic plants caused inhibition of primary growth (e.g., height growth and number of internodes) under drought and/or SDs. In contrast, the transgenic GA modulation had an impact on secondary growth (e.g., stem diameter growth) only under drought but not SDs. In addition, some genotype-specific response differences were found among the transgenics under the two conditions. Most notably, under drought conditions only GA-insensitive (and not GA-deficient) transgenics had significantly reduced relative growth as compared to WT plants (Figure 3A) (discussed below); whereas, under SD conditions all transgenic types had significantly reduced relative growth (Figure 3B).

In addition to growth cessation, reduction of bioactive GA levels and GA signaling in poplar transgenics significantly modified and/or delayed the onset of several other physiological changes that are typically associated with stress response. For instance, stressful conditions typically cause a reduction in photosynthesis and promote leaf senescence, which in turn negatively affects productivity [43]. Thus, delayed leaf senescence and the ability to sustain high photosynthetic capacity in adverse conditions are highly desirable traits [51]. Under drought stress, GA-deficient and GA-insensitive transgenics sustained higher rates of photosynthesis (Table 2). Although we did not measure the photosynthetic rate during SD-induced dormancy, we did observe a delay in leaf senescence in transgenic plants (Figure 6), which is suggestive of a similar response. In support of our findings, GAs have been implicated in the repression of light-regulated genes [52]. Decreases in GA levels via up-regulation of *GA2ox* genes is associated with increases in the expression of light-regulated genes [52], photosynthesis [53], chlorophyll content, and light harvesting chlorophyll proteins [54]. It has also been shown that DELLA proteins are directly involved in photomorphogenesis [55]. In GA-deficient and GA-insensitive transgenics,sustained increases in photosynthesis coupled to reduced shoot growth demands could facilitate shunting of resources from primary to secondary/storage metabolism and/or investment in root growth. Previously, we have shown experimental evidence supporting these hypotheses. For example, the same transgenics also accumulate increased levels of secondary metabolites [36] and show increased levels of root production [36], [37], [40].

In addition, carotenoids are antioxidants that can increase stress resistance via detoxification of ROS [56], and are precursors to the stress-responsive hormone ABA [57]. In Arabidopsis, stress induced DELLA protein accumulation and increased activity of ROS-detoxification enzymes led to reductions in ROS levels [25]. Our microarray data also showed enrichment of GO categories associated with stress response (Table S2, GO:0006950) in the GA-deficient and GA-insensitive transgenics. A number of genes encoding enzymes associated with detoxification of ROS were found in this category, including peroxidases, glutathione-S-transferase, superoxide dismutase, glyoxylate reductase, monodehydroascorbate reductase (Table S1 and 2). Furthermore, genes associated with ABA biosynthesis, such as nine-cis-epoxycarotenoid dioxygense 3, were also highly up-regulated. In summary, DELLA proteins and GA2oxs likely not only repress growth but enhance plant resistance to stress via activation of ROS-detoxification enzymes, increased carotenoid production and ABA biosynthesis.

To better understand the effects of overexpressing GA2ox and DELLA protein encoding genes on drought response, we must also consider the implications of GA-deficient and GA-insensitive transgenics’ semidwarf phenotype. Soil drying experiments can be difficult to interpret because the plants dictate the severity of stress by controlling the balance of water uptake by roots and loss through leaves [41]. Though we implemented methods to minimize genotypic differences in stress severity, semidwarfism could result in plants experiencing a reduced severity of stress. A smaller plant may require less water and thus deplete soil moisture slower. However, it is unlikely that our results are solely reflective of disparities in soil drying causing different stress severities intransgenic and WT plants. If transgenics had experienced a reduced stress severity then we would have expected relative growth rates, similar to those experienced under well-watered conditions, to be sustained longer and delays in growth cessation as compared to WT. However, our results are quite contrary as under drought conditions GA-insensitive transgenics decreased growth much earlier while GA-deficient plants did not differ compared to WT (Figure 3A). Hence, transgenic responses may be due to increased perception of water deficits, morphological differences, and/or altered drought resistant mechanisms.

Because GA-insensitive transgenics responded significantly earlier to water deprivation as compared to WT (Figure 3A), it is unlikely that the hypersensitive drought response induced by GA-insensitivity is a result of increased water availability due to disparities in soil drying. Nevertheless, unlike GA-deficient transgenics, GA-insensitive transgenics had a prompt response to water withdrawal associated with aboveground growth inhibition and suggestive of enhanced drought avoidance mechanisms (Figure 3A). However, the leaves of GA-insensitive transgenics were similar in size but had increased gas exchange when compared to WT (Table 2). Thus, the prompt drought response was likely not a result of decreased leaf water loss. This is further supported by the fact that despite their smaller leaf size, GA-deficient transgenics did not differ from WT in respect to their growth inhibition (Figure 3A). Thus, it is more likely that GA-insensitivity caused transgenic plants to have an enhanced ability to perceive and respond to water deficits. This is strongly supported by a large body of evidence suggesting that the DELLA proteins that cause GA-insensitivity are central hubs for responding to various stresses [23]–[25]. Furthermore under drought stress, leaves of GA-insensitive transgenics were still actively assimilating carbon with increases in photosynthesis and transpiration as compared to WT (Table 2 and Figure S3). The assimilated carbon could be used towards enhancing tolerance mechanisms to protect against cellular damage (osmotic solutes, and ROS detoxification) or to increase root growth, which would be advantageous to water uptake and further promote drought avoidance. Indeed, GA-insensitivity has been previously found to be associated with increased ratios of root mass to leaf area which have been suggested could help sustain higher transpiration rates and decreases in water use efficiency [40]. In summary, the GA-modifications caused complex responses that likelycontribute to both drought avoidance and tolerance mechanisms.

Similar to recent findings by Mauriat et al. [58], we show that the *GA2ox* expressing transgenics have increased branch production which is indicative of decreased apical dominance (Figure 8). However, in contrast to this previous finding, in the present study, the increased branching phenotype developed only after winter dormancy. This difference could be attributed to the different promoters, genetic backgrounds or growing conditions used in this study (note sylleptic branching is very sensitive to environmental quality). It appears that the observed reduction of apical dominance caused by GA2ox up-regulation is a result of decreased auxin concentration and transport [58]. We also show that up-regulation of DELLA proteins leads to a similar phenotype, suggesting that the effect of GA on apical dominance, through modulation of auxin biosynthesis and transport, is downstream of and mediated through a DELLA signaling hub. However, in the DELLA transgenics, branch outgrowth after dormancy was very short-lived and resulted in a very short, stubby branch. In contrast, GA2ox transgenic plants produced very well developed branches from almost all axillary buds. This suggests that DELLA proteins have differential effects on apical dominance before and after dormancy release. Furthermore, this would imply that apical dominance before and after dormancy may be regulated via different branches of the GA signaling pathway or by a completely different mechanism involving different hormones and regulators. These two phenotypic differences in the GA2ox and DELLA transgenics had a significant and dramatic impact on crown shape and size in the field (e.g., wide, ball-shape in GA2ox; narrow compact in the DELLA transgenics) [39].

## Conclusions

In summary, we found that GA catabolism and repressive signaling are part of a highly interactive mechanism for sensing and responding to immediate (drought) and imminent (SD signaling approaching winter) conditions involving ceasing or reducing growth as well as increasing physiologically acclimative responses to stress. These findings suggest that regulation of GA metabolism and response may be a focal point for evolution of various adaptive strategies in response to short-term and prolonged unfavorable conditions. It also suggests novel venues to engineer and breed for improved stress resistance in crop plants.

## Materials and Methods

### Plant Material

Genetic background for all transgenic plants was the INRA 717-1B clone (*Populus tremula* x *P. alba*). For consistency, all experiments involving WT plants were performed using this same genotype. Generation of *35S::PcGA2ox*, *pGAI::gai*, and *35S::rgl1* transgenics was previously described [17], [36]. The Arabidopsis (*Arabidopsis thaliana)* DELLA genes *gai* and *rgl1* have complete truncations of the DELLA domains which confers a gain-of-function dominant mutation with constitutive repression of GA signaling. The *gai* gene was under the control of the native Arabidopsis promoter (pGAI) while *rgl1* was under the control of the cauliflower mosaic virus 35S promoter (35S). The *GA2ox1* gene was from *Phaseolus coccineus* (*PcGA2ox*) and was also under the control of the 35S promoter. Because constructs were previously well-characterized with respect to transgene presence, expression, GA content and stability of phenotype over many years [37], [39], for each transgenic type one representative line with multiple ramets was selected for use in subsequent studies. The differences in the levels of expression of these transgenes in independent transformation events elicits a gradient of phenotypic responses ranging from severe dwarfism to nearly wild-type like [39]. To avoid confounding effects in severely affected plants, we selected lines with intermediate (semi-dwarf) phenotypes.

### Experimental Design

The drought experiment consisted of four genotypes in a completely randomized block design with eight replications. Each block represented a rectangular pot (height = 32 cm, length = 50 cm, and width = 35 cm) assigned four plants: one from each of three transgenic genotypes (*35S::PcGA2ox*, *pGAI::gai*, and *35S::rgl1*) and a WT (untransformed control). Plants were randomized and spaced in a rectangular pattern (25×20 cm apart from each other) within pots. The confiding nature of the pots, allowed plants to be grown in a proximity where their roots were exposed to similar conditions to minimize effects of any differences in water usage between genotypes [41]. The use of relatively large, deep pots facilitated a slow, gradual soil drying process (Figure S2) that provided plants sufficient time for lengthy responses. Plants used in the experiment were propagated and grown in vitro for one month prior to their transfer to a greenhouse. In the greenhouse, plants were placed in a mixture of peat, top soil, perlite, and vermiculite (4∶1∶1∶1, v/v) and grown for approximately two months prior to experiment implementation. The drought treatment consisted of three weeks of well-watered conditions (daily watering regime), followed by a drought stress imposed by completely withholding water for five weeks. A similar experimental regime was used on control plants used in expression analysis (Figure 1) with the exception that plants were grown in a completely randomized design (without blocks), in separate pots, and in a growth chamber (Conviron, Pembina, ND, USA).

SD photoperiod experiments were performed in a completely randomized design with eight replications. Propagation and acclimation of plants to the greenhouse were performed as described above for the drought experiment. After two months of greenhouse growth, plants were transferred to a growth chamber (Conviron, Pembina, ND, USA), with three weeks under LDs (16 h light/8 h dark, at 21°C) followed by six weeks of SDs (8 h light/16 h dark, at 21°C). Plants were then transferred to a cold room (4°C) for 11 weeks to fulfill the chilling requirement needed for resumption of growth. Finally, plants were transferred to LD conditions and bud flush was monitored on a daily basis.

### Biometric and Physiological Measurements

Phenotypic measurements were made on a weekly basis. To avoid problems with soil level changes, all height measurements were made from permanent markers at the base of the plant stem. Net photosynthesis rate, transpiration rate, and stomatal conductance were measured with the portable photosynthesis system LI-6400 (LI-COR, Lincoln, NE, USA), using an air temperature of 25°C and photosynthetically active radiation of 1500 µmol m^−2^ s^−1^. Instantaneous water use efficiency was calculated as net photosynthesis rate dividing by transpiration rate. Gas exchange measurements were made between 8∶00 and 10∶00 am weekly and at leaf plastochron index (LPI) = 10±1. After five weeks of withholding water, additional measurements were made including percent leaf senescence, percent leaf wilt, and electrolyte leakage (EL). EL was measured with a conductivity meter (Model 32, Yellow Springs Instrument Inc., Yellow Springs, OH, USA) using procedures modified from Ren et al. [59] and Verslues et al. [41]. Leaves (LPI = 10±1) were thoroughly washed in distilled water and three 1 cm^2^ discs obtained from leaves were placed in tubes containing 15 ml of distilled water. Tubes were gently agitated for 4 h and initial conductivity was measured. Samples were then autoclaved for 15 min and conductivity was again measured. EL measurements were repeated three times per plant, and values were averaged and expressed as percentage of initial conductivity. Chlorophyll and carotenoid content was measured on non-stressed (well-watered) plants subjected to drought stress (after withholding water for five weeks). Pigments were extracted in N,N-dimethylformamide according to Porra et al. [60]. Pigments were quantified with a spectrophotometer using equations from Porra et al. [60] for chlorophyll a and b, and from Wellburn et al. [61] for carotenoid. Two 1 cm^2^ discs were measured for each plant from LPI = 8±1. Methods similar to those in [62] were used to quantify the relative intensity of green in senescing leaves. Digital images of leaf surfaces were processed using ImageJ version 1.43 (http://rsbweb.nih.gov/ij/) and the Threshold Colour plugin (http://www.dentistry.bham.ac.uk/landinig/software/software.html).

### Expression Analysis

All samples used in expression analysis were collected at the same time weekly, immediately frozen in liquid nitrogen, and stored at −80°C for further use. Samples were analyzed in three biological replications, each consisting of tissues pooled from two to three plants. Total RNA was isolated using the RNeasy Plant Mini Kit (Qiagen,Valencia, CA, USA) with an on-column DNA digestion using RNase-Free DNase (Qiagen). cDNA was synthesized from 2 µg of DNaseI-treated total RNA using Superscript III reverse transcriptase (Invitrogen, Carlsbad, CA, USA) with an oligo-dT primer. One µl of RT reaction was used for RT-PCR. Primers used in the expression analyses are shown in Table 1.

### Statistical Analysis

Statistical analysis was performed using SAS 9.1 (SAS Institute Inc., Cary, NC, USA). In transgenic experiments, treatments consisted of the four genotypes under investigation. For both experiments, analysis of variance (ANOVA) was used to test for overall treatment effects (α = 0.05), and separate ANOVAs were done for each week of the experiment. When significant differences were present, Tukey’s Honestly Significant Difference (HSD) was used to make comparisons among WT and transgenics, or Dunnett’s test (P<0.05) was used when we were interested in making comparison to WT only. For analyses of growth-related parameters, the response variable is the weekly relative growth rate, calculated as: Y = (X_n+1_−X_n_)/X_n_, where Y is weekly relative growth rate, X is the measured growth parameter, and n is the week.

### Microarray Hybridization and Data Analyses

We used a total of three individual genotypes as follows: WT, *35S:PcGA2ox* and *35S:rgl1*. Leaves from two independent biological replicates per genotype were used, each pooled from twenty clonally propagated plants. RNA was isolated as previously described using the Qiagen RNeasy Plant Kit [17]. Prior to labeling, RNA quality was assessed by Agilent Bioanalyzer (Agilent Technologies, Santa Clara, USA) and three µg of total RNA was used to prepare biotinylated complementary RNA (cRNA). The labeling, hybridization, and imaging procedures were performed according to Affymetrix protocols at the Center for Genomics Research and Biocomputing, Oregon State University), using the Affymetrix Poplar GeneChip (Affymetrix Santa Clara, CA, USA). Collection and analysis of data were compliant with MIAME standards (Brazma et al., 2001). Data were analyzed using TM4:MeV [63], [64]. Raw data was first normalized using RMA algorithm [65]. One-way ANOVA (P<0.01) was used to isolate differentially expressed genes. Gene ontology (GO) analyses for significant enrichments of various categories were performed using GOEAST [66] and the corresponding AGI loci using default parameters. Data for trancriptome changes during drought and dormancy were derived from previously published studies [46], [47]. Because of the differences in the microarray platforms and genome versions used in these studies, we used the AGI of the closest Arabidopsis ortholog to compare the three data sets. We have previously validated the results from the array analysis using RT-PCR for 12 genes (six up-regulated and six down-regulated) for the roots from the same plants [37]. The microarray data have been deposited in the Gene Expression Omnibus (GEO) database as accession number GSE38390.

## Supporting Information

Figure S1 **Representative phenotypes and leaf measurements of GA-modified transgenic poplar.** (**A**) Transgenic (*35S::PcGA2ox*; top left, *pGAI::gai*; bottom left, and 35S*::rgl1*; bottom right) and wild-type (WT; top right) plants grown in pots (25×20 cm apart from each other) for three weeks under well-watered conditions, prior to being subjected to five weeks under water-withholding conditions. (**B**) Bars indicate mean±SE of total area (cm^3^) of at least eight mature leaves per a genotype. (**C**) Leaf expansion (cm^3^) was measured weekly under well-watered (week 0) and water-withholding conditions (weeks 1 to 5) on eight ramets/line and eight WT plants. Measurements in **C** represent the total area of expansion of the first unfurled leaf (leaf plastrochon index 1) after one week. Leaf measurements were made from digital images in ImageJ version 1.43 (http://rsbweb.nih.gov/ij/). Significant differences between transgenic and WT plants were determined by one-way ANOVA followed by Dunnett’s post-hoc test (*, P<0.05). Scale bar = 25 cm (**A**).
(TIF)

Figure S2 **Estimation of volumetric soil moisture content (g cm^−3^) in pots under well-water (week 0) and water-withholding conditions (weeks 1 to 5).** A soil auger was used to sample whole vertical profiles of pots. Samples were oven dried at 100°C for at least 24 hrs. Values are means±SE of at least three samples taken from at least two pots.
(TIF)

Figure S3 **Weekly responses of transgenic and WT** ***Populus*** **under well-watered and water- withholding conditions.** The dotted line denotes the initiation of water withholding. Red lines show significant differences between weekly responses of transgenics and WT (see Material and Methods), as determined by one-way ANOVA followed by Dunnett (post-hoc test (P<0.05).
(TIF)

Table S1 **Differential expressed genes in GA-modified transgenics compared to WT poplar.**
(XLSX)

Table S2 **Gene ontology analyses of genes differentially regulated in the GA-modified transgenics.**
(XLSX)

Table S3 **Gene ontology analyses of genes regulated by the GA modifications, drought and winter dormancy.**
(XLSX)

## References

[1] Vinocur B, Altman A (2005) Recent advances in engineering plant tolerance to abiotic stress: achievements and limitations. Curr Opin Biotechnol 16: 123–132. 15831376 10.1016/j.copbio.2005.02.001

[2] Bhatnagar-Mathur P, Vadez V, Sharma KK (2008) Transgenic approaches for abiotic stress tolerance in plants: retrospect and prospects. Plant Cell Rep 27: 411–424. 18026957 10.1007/s00299-007-0474-9

[3] Oerke EC, Dehne HW (2004) Safeguarding production losses in major crops and the role of crop protection. Crop Prot 23: 275–285.

[4] Vasil IK (1998) Biotechnology and food security for the 21st century: a real-world perspective. Nat Biotechnol 16: 399–400. 9592375 10.1038/nbt0598-399

[5] Burke EJ, Brown SJ, Christidis N (2006) Modeling the recent evolution of global drought and projections for the twenty-first century with the hadley centre climate model. J Hydrometeoro 7: 1113–1125.

[6] Ashraf M, Wu L (1994) Breeding for salinity tolerance in plants. Crit Rev Plant Sci 13: 17–42.

[7] Arrigoni O, De Tullio MC (2000) The role of ascorbic acid in cell metabolism: between gene-directed functions and unpredictable chemical reactions. J Plant Physiol 157: 481–488.

[8] Hoffman AA, Parson PA (1993) Evolutionary Genetics and Environmental Stress: Oxford University Press.

[9] Vaughton G, Ramsey M (2001) Variation in summer dormancy in the lilioid geophyte Burchardia umbellata (Colchicaceae). Am J Bot 88: 1223–1229. 11454622

[10] Rohde A, Bhalerao RP (2007) Plant dormancy in the perennial context. Trends Plant Sci 12: 217–223. 17416545 10.1016/j.tplants.2007.03.012

[11] Olszewski N, Sun TP, Gubler F (2002) Gibberellin signaling: biosynthesis, catabolism, and response pathways. Plant Cell 14 Suppl: S61–80 10.1105/tpc.010476 PMC151248 12045270

[12] Hedden P, Thomas SG (2012) Gibberellin biosynthesis and its regulation. Biochem J 444: 11–25. 22533671 10.1042/BJ20120245

[13] Tanimoto E (2012) Tall or short? Slender or thick? A plant strategy for regulating elongation growth of roots by low concentrations of gibberellin. Ann Bot 110: 373–381. 22437663 10.1093/aob/mcs049 PMC3394641

[14] Thomas SG, Phillips AL, Hedden P (1999) Molecular cloning and functional expression of gibberellin 2- oxidases, multifunctional enzymes involved in gibberellin deactivation. Proc Natl Acad Sci USA 96: 4698–4703. 10200325 10.1073/pnas.96.8.4698 PMC16395

[15] Lo SF, Yang SY, Chen KT, Hsing YI, Zeevaart JA, et al (2008) A novel class of gibberellin 2-oxidases control semidwarfism, tillering, and root development in rice. Plant Cell 20: 2603–2618. 18952778 10.1105/tpc.108.060913 PMC2590730

[16] Rieu I, Eriksson S, Powers SJ, Gong F, Griffiths J, et al (2008) Genetic analysis reveals that C19-GA 2-oxidation is a major gibberellin inactivation pathway in Arabidopsis. Plant Cell 20: 2420–2436. 18805991 10.1105/tpc.108.058818 PMC2570722

[17] Busov VB, Meilan R, Pearce DW, Ma C, Rood SB, et al (2003) Activation tagging of a dominant gibberellin catabolism gene (GA 2-oxidase) from poplar that regulates tree stature. Plant Physiol 132: 1283–1291. 12857810 10.1104/pp.103.020354 PMC167068

[18] Gou J, Ma C, Kadmiel M, Gai Y, Strauss S, et al (2011) Tissue-specific expression of Populus C19 GA 2-oxidases differentially regulate above- and below-ground biomass growth through control of bioactive GA concentrations. New Phytol 192: 626–639. 21819406 10.1111/j.1469-8137.2011.03837.x

[19] Fleet CM, Sun TP (2005) A DELLAcate balance: the role of gibberellin in plant morphogenesis. Curr Opin Plant Biol 8: 77–85. 15653404 10.1016/j.pbi.2004.11.015

[20] Dill A, Jung HS, Sun TP (2001) The DELLA motif is essential for gibberellin-induced degradation of RGA. Proc Natl Acad Sci USA 98: 14162–14167. 11717468 10.1073/pnas.251534098 PMC61185

[21] Wen CK, Chang C (2002) Arabidopsis RGL1 encodes a negative regulator of gibberellin responses. Plant Cell 14: 87–100. 11826301 10.1105/tpc.010325 PMC150553

[22] Itoh H, Shimada A, Ueguchi-Tanaka M, Kamiya N, Hasegawa Y, et al (2005) Overexpression of a GRAS protein lacking the DELLA domain confers altered gibberellin responses in rice. Plant J 44: 669–679. 16262715 10.1111/j.1365-313X.2005.02562.x

[23] Achard P, Cheng H, De Grauwe L, Decat J, Schoutteten H, et al (2006) Integration of plant responses to environmentally activated phytohormonal signals. Science 311: 91–94. 16400150 10.1126/science.1118642

[24] Achard P, Gong F, Cheminant S, Alioua M, Hedden P, et al (2008a) The cold-inducible CBF1 factor-dependent signaling pathway modulates the accumulation of the growth-repressing DELLA proteins via its effect on gibberellin metabolism. Plant Cell 20: 2117–2129. 18757556 10.1105/tpc.108.058941 PMC2553604

[25] Achard P, Renou JP, Berthome R, Harberd NP, Genschik P (2008b) Plant DELLAs restrain growth and promote survival of adversity by reducing the levels of reactive oxygen species. Curr Biol 18: 656–660. 18450450 10.1016/j.cub.2008.04.034

[26] Claeys H, Skirycz A, Maleux K, Inze D (2012) DELLA signaling mediates stress-induced cell differentiation in Arabidopsis leaves through modulation of anaphase-promoting complex/cyclosome activity. Plant Physiol 159: 739–747. 22535421 10.1104/pp.112.195032 PMC3375938

[27] Achard P, Gusti A, Cheminant S, Alioua M, Dhondt S, et al (2009) Gibberellin signaling controls cell proliferation rate in Arabidopsis. Curr Biol 19: 1188–1193. 19576768 10.1016/j.cub.2009.05.059

[28] Zentella R, Zhang ZL, Park M, Thomas SG, Endo A, et al (2007) Global analysis of della direct targets in early gibberellin signaling in Arabidopsis. Plant Cell 19: 3037–3057. 17933900 10.1105/tpc.107.054999 PMC2174696

[29] Xiong L, Zhu JK (2003) Regulation of abscisic acid biosynthesis. Plant Physiol 133: 29–36. 12970472 10.1104/pp.103.025395 PMC523868

[30] Magome H, Yamaguchi S, Hanada A, Kamiya Y, Oda K (2008) The DDF1 transcriptional activator upregulates expression of a gibberellin-deactivating gene, GA2ox7, under high-salinity stress in Arabidopsis. Plant J 56: 613–626. 18643985 10.1111/j.1365-313X.2008.03627.x

[31] Yamauchi Y, Ogawa M, Kuwahara A, Hanada A, Kamiya Y, et al (2004) Activation of gibberellin biosynthesis and response pathways by low temperature during imbibition of Arabidopsis thaliana seeds. Plant Cell 16: 367–378. 14729916 10.1105/tpc.018143 PMC341910

[32] Finch-Savage WE, Leubner-Metzger G (2006) Seed dormancy and the control of germination. New Phytol 171: 501–523. 16866955 10.1111/j.1469-8137.2006.01787.x

[33] Olsen JE, Jensen E, Junttila O, Moritz T (1995a) Photoperiodic control of endogenous gibberellins in seedlings of Salix pentandra. Physiol Plant 93: 639–644.

[34] Olsen JE, Junttila O, Moritz T (1995b) A localised decrease of GA1 in shoot tips of Salix pentandra seedings precedes cessation of shoot elongation under short photoperiod. Physiol Plant 95: 627–632.

[35] Junttila O, Jensen E (1988) Gibberellins and photoperiodic control of shoot elongation in Salix. Physiol Plant 74: 371–376.

[36] Busov V, Meilan R, Pearce DW, Rood SB, Ma C, et al (2006) Transgenic modification of gai or rgl1 causes dwarfing and alters gibberellins, root growth, and metabolite profiles in Populus. Planta 224: 288–299. 16404575 10.1007/s00425-005-0213-9

[37] Gou J, Strauss SH, Tsai CJ, Fang K, Chen Y, et al (2010) Gibberellins regulate lateral root formation in Populus through interactions with auxin and other hormones. Plant Cell 22: 623–639. 20354195 10.1105/tpc.109.073239 PMC2861444

[38] Zawaski C, Kadmiel M, Ma C, Gai Y, Jiang X, et al (2011) SHORT INTERNODES-like genes regulate shoot growth and xylem proliferation in Populus. New Phytol 191: 678–691. 21564099 10.1111/j.1469-8137.2011.03742.x

[39] Zawaski C, Kadmiel M, Pickens J, Ma C, Strauss S, et al (2011) Repression of gibberellin biosynthesis or signaling produces striking alterations in poplar growth, morphology, and flowering. Planta 234: 1285–1298. 21792553 10.1007/s00425-011-1485-x

[40] Elias AA, Busov VB, Kosola KR, Ma C, Etherington E, et al (2012) Green Revolution Trees: Semidwarfism Transgenes Modify Gibberellins, Promote Root Growth, Enhance Morphological Diversity, and Reduce Competitiveness in Hybrid Poplar. Plant Physiology 160: 1130–1144. 22904164 10.1104/pp.112.200741 PMC3461535

[41] Verslues PE, Agarwal M, Katiyar-Agarwal S, Zhu J, Zhu JK (2006) Methods and concepts in quantifying resistance to drought, salt and freezing, abiotic stresses that affect plant water status. Plant J 45: 523–539. 16441347 10.1111/j.1365-313X.2005.02593.x

[42] Rohde A, Prinsen E, De Rycke R, Engler G, Van Montagu M, et al (2002) PtABI3 impinges on the growth and differentiation of embryonic leaves during bud set in poplar. Plant Cell 14: 1885–1901. 12172029 10.1105/tpc.003186 PMC151472

[43] Faver KL, Gerik TJ, Thaxton PM, El-Zik KM (1996) Late season water stress in cotton: Ii. leaf gas exchange and assimilation capacity. Crop Sci 36: 922–928.

[44] Percival GC, Fraser GA (2001) Measurement of the salinity and freezing tolerance of Crataegus genotypes using chlorophyll fluorescence. J Arboric 27: 233–245.

[45] Weiss D, Ori N (2007) Mechanisms of cross talk between gibberellin and other hormones. Plant Physiol 144: 1240–1246. 17616507 10.1104/pp.107.100370 PMC1914132

[46] Ruttink T, Arend M, Morreel K, Storme V, Rombauts S, et al (2007) A molecular timetable for apical bud formation and dormancy induction in poplar. Plant Cell 19: 2370–2390. 17693531 10.1105/tpc.107.052811 PMC2002631

[47] Wilkins O, Waldron L, Nahal H, Provart NJ, Campbell MM (2009) Genotype and time of day shape the Populus drought response. Plant J 60: 703–715. 19682285 10.1111/j.1365-313X.2009.03993.x

[48] Tran LS, Nakashima K, Shinozaki K, Yamaguchi-Shinozaki K (2007) Plant gene networks in osmotic stress response: from genes to regulatory networks. Methods Enzymol 428: 109–128. 17875414 10.1016/S0076-6879(07)28006-1

[49] Smith H (2000) Phytochromes and light signal perception by plants–an emerging synthesis. Nature 407: 585–591. 11034200 10.1038/35036500

[50] Achard P, Genschik P (2009) Releasing the brakes of plant growth: how GAs shutdown DELLA proteins. J Exp Bot 60: 1085–1092. 19043067 10.1093/jxb/ern301

[51] Ortiz R (2008) Crop genetic engineering under global climate change. Ann Arid Zone 47: 1–12.

[52] Alabadi D, Gil J, Blazquez MA, Garcia-Martinez JL (2004) Gibberellins repress photomorphogenesis in darkness. Plant Physiol 134: 1050–1057. 14963246 10.1104/pp.103.035451 PMC389929

[53] Biemelt S, Tschiersch H, Sonnewald U (2004) Impact of altered gibberellin metabolism on biomass accumulation, lignin biosynthesis, and photosynthesis in transgenic tobacco plants. Plant Physiol 135: 254–265. 15122040 10.1104/pp.103.036988 PMC429367

[54] Mathis JN, Bradburne JA, Dupree MA (1989) Gibberellic Acid effects on greening in pea seedlings. Plant Physiol 91: 19–22. 16666994 10.1104/pp.91.1.19 PMC1061944

[55] Achard P, Liao L, Jiang C, Desnos T, Bartlett J, et al (2007) DELLAs Contribute to Plant Photomorphogenesis. Plant Physiol 143: 1163–1172. 17220364 10.1104/pp.106.092254 PMC1820925

[56] DellaPenna D, Pogson BJ (2006) Vitamin synthesis in plants: tocopherols and carotenoids. Annu Rev Plant Biol 57: 711–738. 16669779 10.1146/annurev.arplant.56.032604.144301

[57] Fang J, Chai C, Qian Q, Li C, Tang J, et al (2008) Mutations of genes in synthesis of the carotenoid precursors of ABA lead to pre-harvest sprouting and photo-oxidation in rice. Plant J 54: 177–189. 18208525 10.1111/j.1365-313X.2008.03411.x PMC2327239

[58] Mauriat M, Sandberg LG, Moritz T (2011) Proper gibberellin localization in vascular tissue is required to control auxin-dependent leaf development and bud outgrowth in hybrid aspen. Plant J 67: 805–816. 21569133 10.1111/j.1365-313X.2011.04635.x

[59] Ren J, Yao Y, Yang Y, Korpelainen H, Junttila O, et al (2006) Growth and physiological responses to supplemental UV-B radiation of two contrasting poplar species. Tree Physiol 26: 665–672. 16452080 10.1093/treephys/26.5.665

[60] Porra RJ, Thompson WA, Kriedemann PE (1989) Determination of accurate extinction coefficients and simultaneous equations for assaying chlorophylls a and b extracted with four different solvents: verification of the concentration of chlorophyll standards by atomic absorption spectroscopy. Biochimica et Biophysica Acta 975: 384–394.

[61] Wellburn AR (1994) The Spectral determination of chlorophylls a and b, as well as total carotenoids, using various solvents with spectrophotometers of different resolution. J Plant Physiol 144: 307–313.

[62] Bouvier-Nave P, Berna A, Noiriel A, Compagnon V, Carlsson AS, et al (2010) Involvement of the phospholipid sterol acyltransferase1 in plant sterol homeostasis and leaf senescence. Plant Physiol 152: 107–119. 19923239 10.1104/pp.109.145672 PMC2799350

[63] Saeed AI, Sharov V, White J, Li J, Liang W, et al (2003) TM4: a free, open-source system for microarray data management and analysis. Biotechniques 34: 374–378. 12613259 10.2144/03342mt01

[64] Chu VT, Gottardo R, Raftery AE, Bumgarner RE, Yeung KY (2008) MeV+R: using MeV as a graphical user interface for Bioconductor applications in microarray analysis. Genome Biol 9: R118. 18652698 10.1186/gb-2008-9-7-r118 PMC2530872

[65] Bolstad BM, Irizarry RA, Astrand M, Speed TP (2003) A comparison of normalization methods for high density oligonucleotide array data based on variance and bias. Bioinformatics 19: 185–193. 12538238 10.1093/bioinformatics/19.2.185

[66] Zheng Q, Wang XJ (2008) GOEAST: a web-based software toolkit for Gene Ontology enrichment analysis. Nucleic Acids Res 36: W358–363. 18487275 10.1093/nar/gkn276 PMC2447756

