# Supplementary figures and images for: Roles of Gibberellin Catabolism and Signaling in Growth and Physiological Response to Drought and Short-Day Photoperiods in Populus Trees
Source: PLoS One. 2014 Jan 20;9(1):e86217. doi: 10.1371/journal.pone.0086217 (PMC3896445; doi:10.1371/journal.pone.0086217)

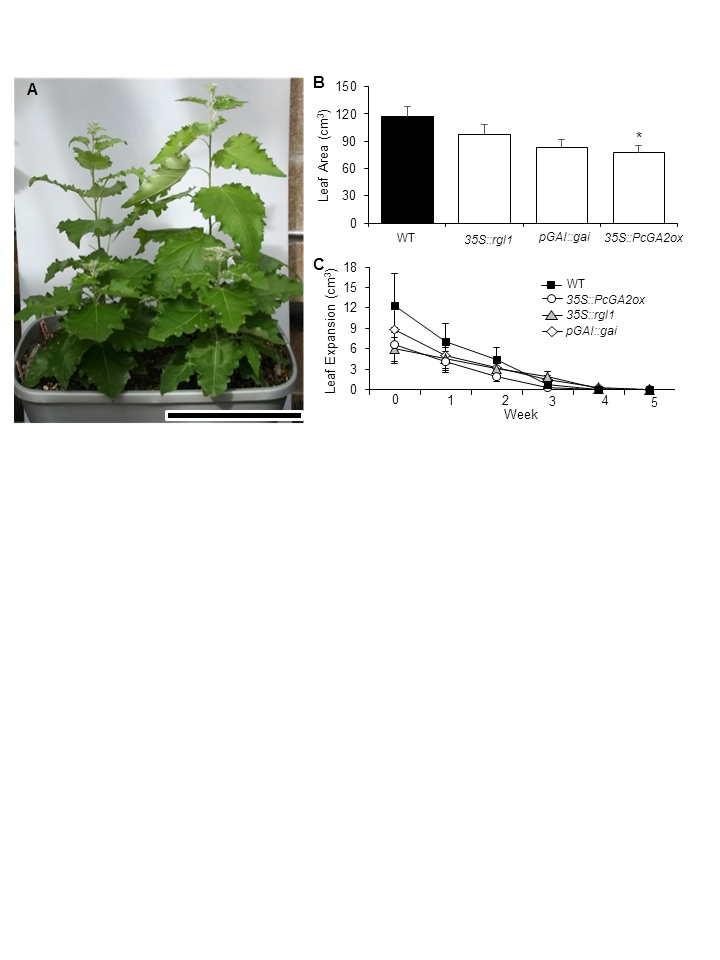

Supplement: Figure S1 — Representative phenotypes and leaf measurements of GA-modified transgenic poplar. (A) Transgenic (35S::PcGA2ox; top left, pGAI::gai; bottom left, and 35S::rgl1; bottom right) and wild-type (WT; top right) plants grown in pots (25×20 cm apart from each other) for three weeks under well-watered conditions, prior to being subjected to five weeks under water-withholding conditions. (B) Bars indicate mean±SE of total area (cm3) of at least eight mature leaves per a genotype. (C) Leaf expansion (cm3) was measured weekly under well-watered (week 0) and water-withholding conditions (weeks 1 to 5) on eight ramets/line and eight WT plants. Measurements in C represent the total area of expansion of the first unfurled leaf (leaf plastrochon index 1) after one week. Leaf measurements were made from digital images in ImageJ version 1.43 (http://rsbweb.nih.gov/ij/). Significant differences between transgenic and WT plants were determined by one-way ANOVA followed by Dunnett’s post-hoc test (*, P<0.05). Scale bar = 25 cm (A). (TIF) [file pone.0086217.s001.tif]

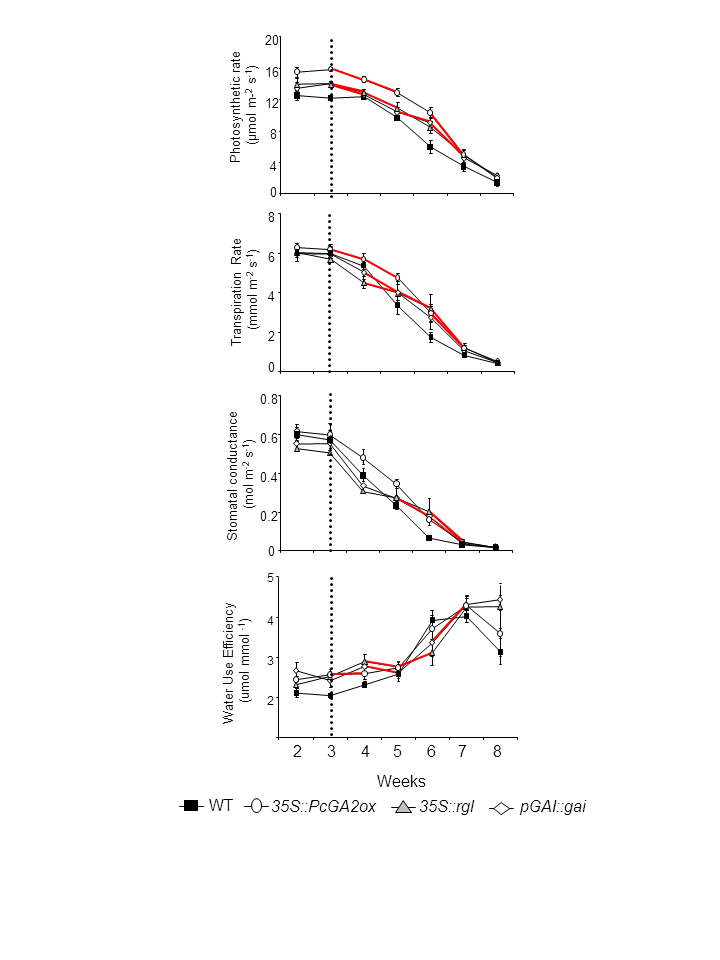

Supplement: Figure S3 — Weekly responses of transgenic and WT Populus under well-watered and water- withholding conditions. The dotted line denotes the initiation of water withholding. Red lines show significant differences between weekly responses of transgenics and WT (see Material and Methods), as determined by one-way ANOVA followed by Dunnett (post-hoc test (P<0.05). (TIF) [file pone.0086217.s003.tif]
